# The Role of Transient Receptor Potential Melastatin 7 (TRPM7) in Cell Viability: A Potential Target to Suppress Breast Cancer Cell Cycle

**DOI:** 10.3390/cancers12010131

**Published:** 2020-01-04

**Authors:** Hengrui Liu, James P. Dilger, Jun Lin

**Affiliations:** Department of Anesthesiology, Health Science Center, Stony Brook University, Stony Brook, NY 11794, USA; hengrui.liu1@stonybrookmedicine.edu (H.L.); james.dilger@stonybrookmedicine.edu (J.P.D.)

**Keywords:** trpm7, cell cycle, breast cancer, hek293, 2-aminoethyl diphenylborinate

## Abstract

The divalent cation-selective channel transient receptor potential melastatin 7 (TRPM7) channel was shown to affect the proliferation of some types of cancer cell. However, the function of TRPM7 in the viability of breast cancer cells remains unclear. Here we show that TRPM inhibitors suppressed the viability of TRPM7-expressing breast cancer cells. We first demonstrated that the TRPM7 inhibitors 2-aminoethyl diphenylborinate (2-APB), ginsenoside Rd (Gin Rd), and waixenicin A preferentially suppressed the viability of human embryonic kidney HEK293 overexpressing TRPM7 (HEK-M7) cells over wildtype HEK293 (WT-HEK). Next, we confirmed the effects of 2-APB on the TRPM7 channel functions by whole-cell currents and divalent cation influx. The inhibition of the viability of HEK-M7 cells by 2-APB was not mediated by the increase in cell death but by the interruption of the cell cycle. Similar to HEK-M7 cells, the viability of TRPM7-expressing human breast cancer MDA-MB-231, AU565, and T47D cells were also suppressed by 2-APB by arresting the cell cycle in the S phase. Furthermore, in a novel TRPM7 knock-out MDA-MB-231 (KO-231) cell line, decreased divalent influx and reduced proliferation were observed compared to the wildtype MDA-MB-231 cells. 2-APB and Gin Rd preferentially suppressed the viability of wildtype MDA-MB-231 cells over KO-231 by affecting the cell cycle in wildtype but not KO-231 cells. Our results suggest that TRPM7 regulates the cell cycle of breast cancers and is a potential therapeutic target.

## 1. Introduction

The regulation of intracellular ion homeostasis is critical for the normal cell function of nonexcitable cells. Unlike excitable cells, where ion entry is mediated by voltage-gated ion channels, nonexcitable cells usually lack these channels [1,2,3]. Transient receptor potential (TRP) channels have been thought to regulate intracellular Ca^2+^ homeostasis in nonexcitable cells. There are six subfamilies in the TRP superfamily of ion channels and one of them, the melastatin-like transient receptor potential (TRPM), has eight members, TRPM1 to TRPM8 [4,5,6]. The data suggest that TRPM7 (also known as LTRPC7, ChaK1 and TRP-PLIK) is aberrantly expressed in cancer cells and plays a crucial role in cancer [7], implying a role for TRPM7 as a molecular biomarker and a therapeutic target in human malignancies [8].

TRPM7 is overexpressed and required for proliferation in bladder cancer [9] and breast cancer cells [10]. TRPM7 channels are permeable to both Ca^2+^ and Mg^2+^. As Ca^2+^ is an essential regulator for the cell cycle and proliferation, the function of the Ca^2+^ influx is considered to be one of the mechanisms by which TRPM7 regulates cell viability. The Ca^2+^ influx current mediated by TRPM7 was reported to play an important role in human head and neck carcinoma cell proliferation [11]. Because the permeability of TRPM7 for Mg^2+^ is higher than for Ca^2+^ [12], Mg^2+^ homeostasis regulated by TRPM7 [13,14] might play a more important role. TRPM7 affects magnesium-nucleotide-regulated metal ion currents, which are critical for cell viability [15]. However, the exact role played by TRPM7 in breast cancer cell viability has not been explored.

In this study, we examined the role of TRPM7 channels in the proliferation, cytotoxicity, and apoptosis first in wild type HEK cells (WT-HEK) and a HEK293 cell line overexpressing TRPM7 (HEK-M7), and then in breast cancer cells, in which, for the first time, a novel TRPM7 knock-out MDA-MB-231 cell line is used. Limited by the paucity of ideal specific TRPM7 inhibitors, we employed several non-specific inhibitors to study the TRPM7 channels in the cells. Our data provide further insight regarding the role of TRPM7 channels in the viability of breast cancer cells by the regulation of the cell cycle.

## 2. Results

### 2.1. Effects of TRPM7 Inhibitors on Cell Viability of WT-HEK and HEK-M7

**Figure 1:**
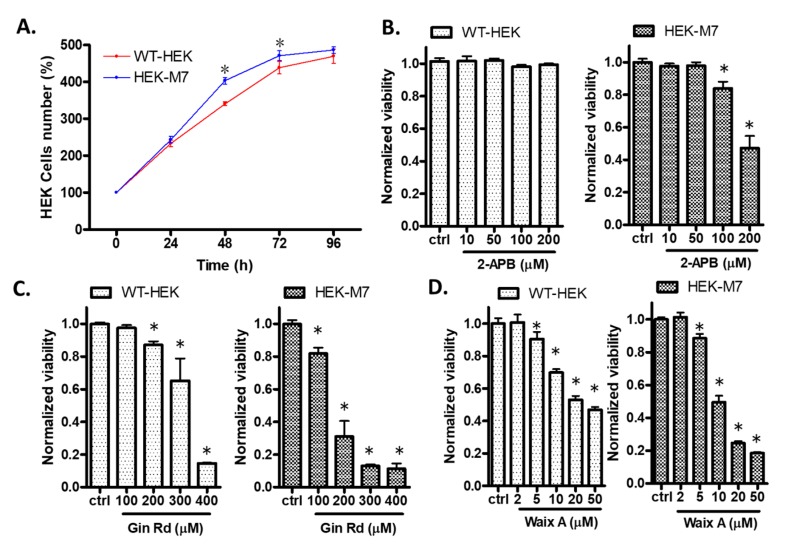
Effect of transient receptor potential melastatin 7 (TRPM7) inhibitors on wildtype human embryonic kidney HEK293 (WT-HEK) and human embryonic kidney HEK293 overexpressing TRPM7 (HEK-M7) cell viability. (**A**) WT-HEK and HEK-M7 cells proliferated similarly at 24. There was a significant difference in the number of cells at 48 and 72 h, but not 96 h. (**B**–**D**) The effect of the TRPM7 inhibitors 2-aminoethyl diphenylborinate (2-APB), ginsenoside Rd (Gin Rd), and waixenicin A (Waix A) on WT-HEK and HEK-M7 viability tested using MTT assay. All three inhibitors tested preferentially inhibited the viability of HEK-M7 over WT-HEK cells. Significant differences (*p* < 0.05) from the control are indicated by “*”.

We monitored the growth of HEK cells in the absence of inhibitors. There was no difference of cell proliferation between WT-HEK and HEK-M7 cells at 24 h. The density of HEK-M7 cells was 18.6% and 7.5% greater than that of WT-HEK cells at 48 and 72 h, respectively. At 96 h, both cell lines had reached confluency (Figure 1A). Therefore, subsequent assays after the incubation of cells with inhibitors were performed at 24 h. We expected that the inhibition of TRPM7 would suppress cell viability. Here, we tested three TRPM7 inhibitors—2-Aminoethyl diphenylborinate (2-APB), Ginsenoside Rd (Gin Rd), and Waixenicin A (Waix A). Based on previous studies [16,17,18], the concentrations we tested for 2-APB were 10–200 µM, for Gin Rd werte 100–400 µM, and for Waix A were 2–50 µM. All three inhibitors preferentially inhibited the viability of HEK-M7 over WT-HEK cells, showing rightward shifts in the dose–response curves (Figure 1B–D). 2-APB has been widely used in the TRPM7 studies [19,20] and inhibited the viability of HEK-M7 but not WT-HEK cells at 100–200 µM (Figure 1B). Therefore, we chose 2-APB (200 µM, 24 h) in subsequent experiments and used a second inhibitor, Gin Rd, to confirm the effect of TRPM7 inhibition in breast cancer cells.

### 2.2. 2-APB Inhibited TRPM7 Current and Divalent Flux

**Figure 2:**
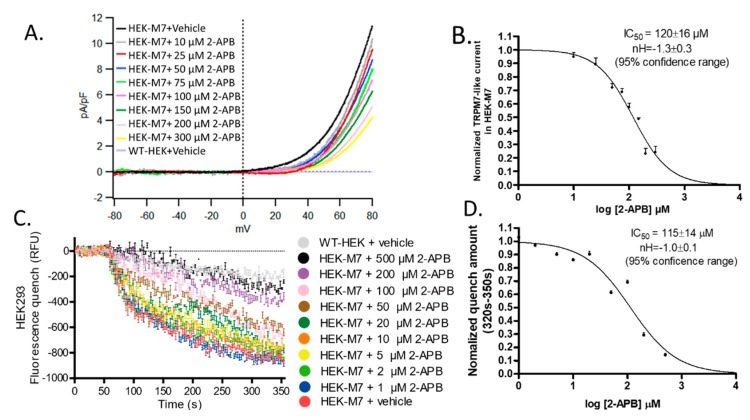
Effect of 2-APB on TRPM7 channels. (**A**) Representative TRPM7-like current from a whole-cell patch-clamp experiment with a HEK-M7 cell. The voltage was clamped at −80 mV, then ramped from −80 to +80 mV. The outwardly rectifying current at positive potentials was suppressed by a perfusion solution containing 2-APB and completely reversed by perfusion with a 2-APB-free solution. There was a very low rectifying current (2.2 fA/pF at + 80 mV) in the WT-HEK cells (Figure S1). (**B**) Normalized current at +80 mV (*n* ≥ 5). The IC_50_ and Hill slope of the current blockade by 2-APB were 120 ± 16 µM and −1.3 ± 0.3, respectively (95% confidence range). (**C**) Results from experiments using Mn^2+^ quenching of Fura-2AM fluorescence. Mn^2+^ and 2-APB were added after 50 s baseline measurement. 2-APB suppressed fluorescence quenching by blocking entry of Mn^2+^ via TRPM7 channels. Fluorescence quenching in WT-HEK cells may reflect the flux of Mn^2+^ through pathways other than TRPM7 channels. (**D**) Normalized quench levels averaged over 320–350 s (*n* = 3). The IC_50_ and Hill slope of quench blockade was 115 ± 14 µM and −1.0 ± 0.1, respectively (95% confidence range).

We examined the TRPM7 channel function with whole-cell patch-clamp recordings. The signature of TRPM7 currents is a strong outward rectification. Endogenous TRPM7 channels have been reported in HEK cells [16,17]. As expected, WT-HEK cells showed little TRPM7-like current (Figure S1), whereas HEK-M7 cells showed a robust TRPM7-like current. This current was concentration-dependently suppressed by 2-APB (Figure 2A). The IC_50_ and Hill slope of the current blockade at +80 mV was 120 ± 16 µM and −1.3 ± 0.3 respectively (95% confidence range) (Figure 2B). Because TRPM7 currents are so small at the physiological voltages, the currents are typically measured at unphysiologically high positive potentials. To confirm that the inhibition by 2-APB is not affected by the potential, we used a fura-2AM-based fluorescence quench assay that reflects the flux of a divalent cation (Mn^2+^) at the cell’s resting potential. Although fluorescence quenching at 300 s after the addition of Mn^2+^ (at 50 s) was detected in WT-HEK, the effect was much greater in HEK-M7 cells. Although we expect some of the flux through WT-HEK cells to come from TRPM7 channels, most of the flux may represent nonspecific flux of divalent cations. In HEK-M7 cells, the fluorescence was concentration-dependently quenched by 2-APB (Figure 2C). The IC_50_ and Hill slope of the average quench amount at 320–350 s was 115 ± 14 µM and −1.0 ± 0.1, respectively (95% confidence range) (Figure 2D). Thus, the potency of 2-APB was the same for both TRPM7 functional assays.

### 2.3. 2-APB Suppressed the Cell Proliferation in HEK-M7 but not WT-HEK Cells

**Figure 3:**
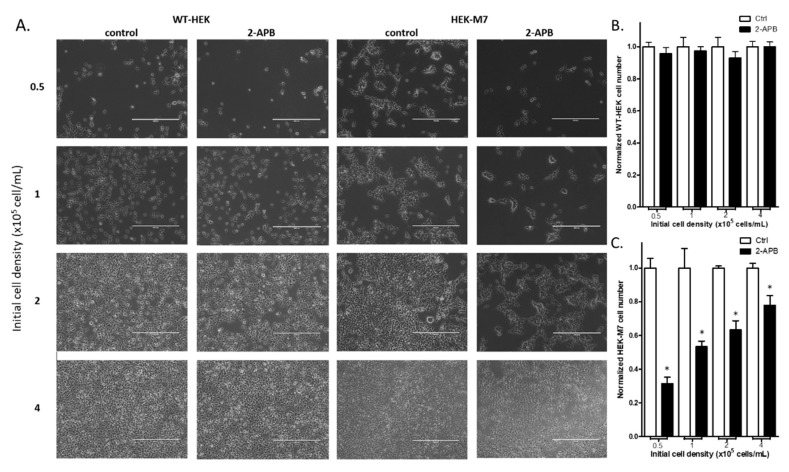
Suppression of cell proliferation in WT-HEK and HEK-M7 by 2-APB. Cells were planted in different cell densities and were treated with 200 µM 2-APB for 24 h. (**A**) Representative images of the cells. The scale bar corresponds to 400 µM. (**B**,**C**) Cell counting assay showed that 2-APB did not change the cell number in WT-HEK, whereas in HEK-M7, there was a density-dependent decrease in cell number. Significant differences (*p* < 0.05) from the control are indicated by “*”.

To confirm the selective inhibition by 2-APB (200 µM) on the proliferation of HEK-M7 over WT-HEK cells, we counted the cell number in both cell lines after a 24-h treatment with 200 µM 2-APB. We examined cells plated at different densities to determine whether inhibition is dependent on cell–cell contact inhibition (proliferation suppression) (Figure 3A). Our results showed that 2-APB did not affect the WT-HEK cell number at any plating density (Figure 3B). However, 2-APB significantly decreased the cell number with HEK-M7 and the magnitude of the suppression decreased when cells were plated at higher densities (Figure 3C).

### 2.4. 2-APB Inhibition of TRPM7 Affected the Cell Cycle but did not Promote Cell Death

**Figure 4:**
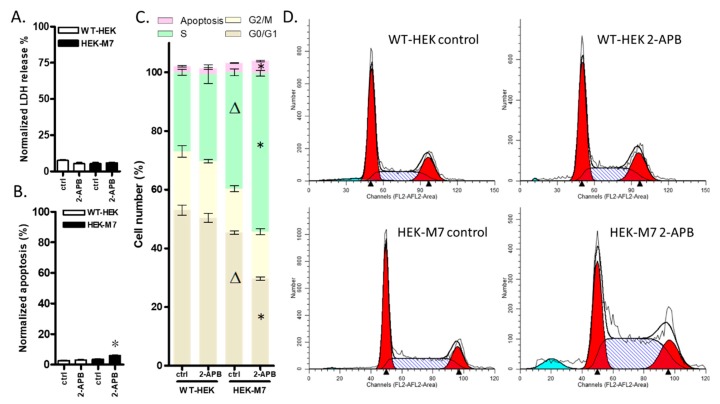
The role of TRPM7 channels in HEK cell biology. (**A**–**D**) show data for cells exposed to 200 µM 2-APB for 24 h. (**A**) lactate dehydrogenase (LDH) Release. 2-APB had no significant effect on LDH release in either WT-HEK or HEK-M7 cells. (**B**) Apoptosis. 2-APB induced a small but significant amount of apoptosis in HEK-M7 only. (**C**) Cell cycle analysis. Overexpression of TRPM7 caused an increase in the percentage of cells in the S phase and a corresponding decrease in the G0/G1 phase in HEK cells. 2-APB caused a further increase in the percentage of cells in the S phase and the corresponding decrease in the G0/G1 phase in HEK-M7 but did not affect the cell cycle of WT-HEK cells. (**D**) Representative images from the cell cycle assay of HEK cell lines. Significant differences (*p* < 0.05) from control and wildtype control are indicated by “*” and “^Δ^” respectively.

To help define the pathways involved in the decrease in HEK-M7 cell viability by 2-APB, we conducted lactate dehydrogenase (LDH), apoptosis and cell cycle assays. The overall viability of cells is the comprehensive reflection of proliferation and cell death. The LDH assay tests the cell damage by the determination of the cell membrane integrity and the apoptosis assay tests the programmed cell death. Our results showed that the LDH levels in both HEK cell lines were about 10% and were not affected by 2-APB (Figure 4A). The overexpression of TRPM7 did not affect cell apoptosis, but 2-APB slightly increased cell apoptosis in HEK-M7 only (Figure 4B). The cell cycle analysis showed that overexpressing TRPM7 caused an 11% increase in the percentage of cells in the S phase and a corresponding decrease in the G0/G1 phase. 200 µM 2-APB did not significantly affect the cell cycle of WT-HEK cells. However, the application of 200 µM 2-APB to HEK-M7 cells caused a further 15% increase in the percentage of cells in the S phase, with a corresponding decrease in the G0/G1 phase and a slight increase in apoptosis (Figure 4C,D).

### 2.5. 2-APB Suppressed Cell Viability in TRPM7-Expressing Breast Cancer Cell Lines

**Figure 5:**
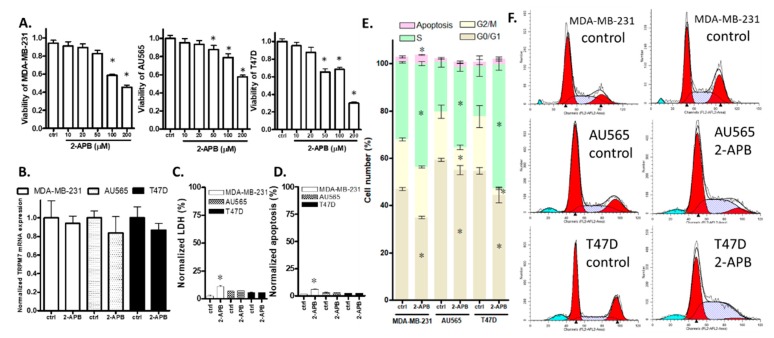
Effects of 2-APB on breast cancer cell lines. (**A**) Cell viability. The 24-h treatment with 2-APB (50–200 µM) significantly suppressed viability in all three cell lines except for 50 µM 2-APB on MDA-MB-231. (**B**–**E**) show data for cells exposed to 200 µM 2-APB for 24 h. (**B**) TRPM7 mRNA levels. 2-APB did not significantly affect TRPM7 mRNA expression. (**C**) LDH release. 2-APB had a significant effect on the LDH of MDA-MB-231 only. (**D**) Apoptosis. 2-APB had a significant effect on the apoptosis of MDA-MB-231 only. (**E**) 2-APB caused an increase in the percentage of cells in the S phase and a corresponding decrease in cells in the G0/G1 phase in all three cell lines and also caused a decrease in the number of cells in the G2/M phase in AU565 and T47D. (**F**) Representative images from the cell cycle assay of breast cancer cell lines. Significant differences (*p* < 0.05) from control are indicated by “*”.

We selected three breast cancer cell lines MDA-MB-231, AU565, and T47D [21] and measured the expression of TRPM7 and the effect of 2-APB on TRPM7 expression in these cells. RT-QPCR results showed that TRPM7 mRNA was expressed in these breast cancer cell lines and that the expression was not significantly changed by a 24-h treatment with 2-APB (Figure 5B). Then, we tested the effect of 2-APB on the viability of these breast cancer cell lines. The MTT assay showed that a 24-h treatment with 100–200 µM 2-APB suppressed the viability of all three breast cancer cell lines, while 50 µM 2-APB suppressed the viability of AU565 and T47D but not MDA-MB-231 (Figure 5A). In subsequent experiments with these cancer cell lines, 200 µM 2-APB was used.

### 2.6. 2-APB Decreased the Overall Viability of AU565 and T47D by Inhibiting Cell Proliferation Rather Than by Promoting Cell Death

To help define the processes involved in the decrease in breast cancer cell viability by 2-APB, we conducted LDH, apoptosis and cell cycle assays. The results of the LDH and apoptosis assays showed that 200 µM 2-APB increased LDH level (Figure 5C) and induced apoptosis (Figure 5D) in MDA-MB-231 cells but not AU565 or T47D cells. Cell cycle analysis showed that a 24-h treatment of cells with 200 µM 2-APB caused an increase in the percentage of cells in the S phase and a decrease in the G0/G1 phase in all the three breast cancer cell lines. In addition, 2-APB decreased the G2/M phase in AU565 and T47D cells. In agreement with the apoptosis assay, cell cycle analysis showed that 2-APB caused a slight increase in apoptosis in MDA-MB-231 cells only (Figure 5E).

### 2.7. The Knock-out of TRPM7 Decreased the Divalent Influx in MDA-MB-231

**Figure 6:**
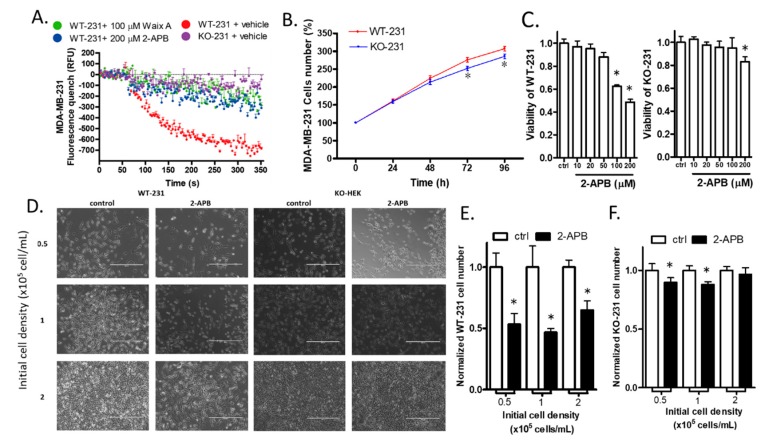
The effect of TRPM7 knockout on MDA-MB-231. (**A**) Mn^2+^ flux. Mn^2+^ and drugs were added after the 50 s baseline measurement. Fura-2AM-based fluorescence quench assay showed that knock-out of TRPM7 in MDA-MB-231 (KO-231) suppressed the function of TRPM7 channels. The block by 2-APB or Waix A in wild type MDA-MB-231 (WT-231) was used as positive controls. (**B**) Cell proliferation. Knock-out of TRPM7 in MBA-MD-231 did not significantly affect cell number within 48 h but decreased cell number after 72 and 96 h. (**C**) Viability. 2-APB (100 µM, which was used in the subsequent experiments) suppressed viability in WT-231 only, while 2-APB (200 µM) significantly suppressed viability in both WT-231 and KO-231, with a higher suppression rate in WT-231 than KO-231. (**D**) Cell number. Representative images from the cell counting experiment of MDA-MB-231 cells. Cells were planted in different cell densities and were treated with 200 µM 2-APB for 24 h. The scale bar corresponds to 400 µm. (**E**,**F**) The cell counting assay showed that 2-APB dramatically decreased the WT-231 cell number in all groups, whereas it only slightly decreased the KO-231 cell number in the 0.5 × 10^5^ and 1 × 10^5^ groups. Significant differences (*p* < 0.05) from the control are indicated by “*”.

The cell line in which TRPM7 was knocked-out from MDA-MB-231 cells provided a way of determining the role of TRPM7 in a breast cancer cell line. We use the abbreviations WT-231 and KO-231 to represent wild-type MDA-MB-231 and TRPM7 knock-out MDA-MB-231, respectively, in the following sections. As expected for TRPM7 knock-out, KO-231 cells had significantly less cation influx than WT-231 cells, as determined by the fluorescence quench assay. The influx through KO-231 cells was significantly less (*p* < 0.01) than that through WT-231 cells exposed to TRPM7 inhibitors 2-APB and Waix A (Figure 6A, Figure S2).

### 2.8. The Knock-out of TRPM7 Decreased the Proliferation of WT-231 Cells

To determine whether knock-out of TRPM7 affects breast cancer cell proliferation, we cultured and counted the WT-231 and KO-231 cells. The cell numbers between WT-HEK and HEK-M7 became significantly different at 72 and 96 h (Figure 6B).

### 2.9. 2-APB Selectively Suppressed the Viability of WT-231 Compared with KO-231

We compared the effect of 2-APB on the viability of WT-231 and KO-231. The MTT assay showed that 100 µM 2-APB suppressed the viability of WT-231 only. Although 200 µM 2-APB did suppress the viability of KO-231 cells by 15%, it suppressed the viability of WT-231 cells by 50% (Figure 6C). Thus, in subsequent experiments, we adopted a 24-h treatment of cells with 100 µM 2-APB. To confirm the inhibition of 2-APB (100 µM) on the viability of WT-HEK over KO-231 cells, we counted the cell number of both cell lines after 24 h of 2-APB treatment. Cells in different densities were counted respectively to see if cell–cell contact inhibition affects the impact of 2-APB (Figure 6D). The cell counting results show that 2-APB significantly decreased the cell number of WT-231 (Figure 6E) but only slightly decreased the KO-231 cell number (Figure 6F).

### 2.10. The Viability of MDA-MB-231 Was Affected by 100 µM 2-APB by Regulating Cell Cycle rather than Cell Death (LDH and Apoptosis) and this Effect was Blocked by TRPM7 Knock-out

**Figure 7:**
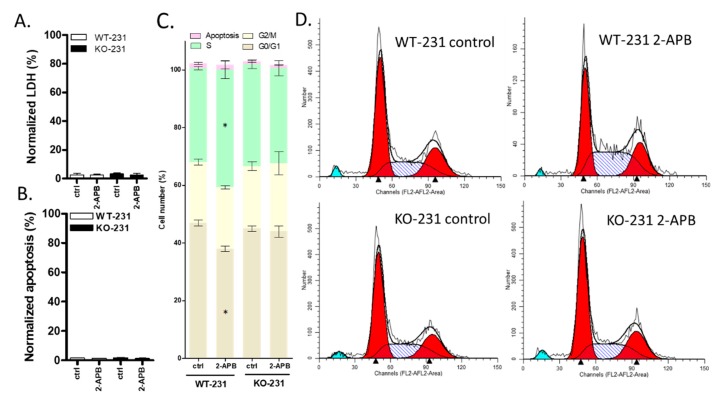
The effect of 2-APB on TRPM7 knockout MDA-MB-231 cell biology. (**A**–**D**) show data for cells exposed to 100 µM 2-APB for 24 h. (**A**) LDH release. 2-APB had no significant effect on LDH. (**B**) Apoptosis. 2-APB had no significant effect on apoptosis in either cell lines. (**C**) Cell cycle analysis. The knock-out of TRPM7 did not affect the cell cycle. 2-APB caused an increase in the percentage of cells in the S phase and a corresponding decrease in the G0/G1 phase in WT-231 but not in KO-231. (**D**) Representative images from the cell cycle assay of MDA-MB-231. Significant differences (*p* < 0.05) from control are indicated by “*”.

To help define the role of TRPM7 in the decrease in WT-231 viability by 2-APB, we conducted LDH, apoptosis and cell cycle assays with both WT-231 and KO-231. LDH and apoptosis assays showed that both the knock-out of TRPM7 and the treatment of 100 µM 2- APB did not affect either the LDH level or apoptosis (Figure 7A,B). The cell cycle analysis showed that the knocking out of TRPM7 did not affect cell cycle, but 100 µM 2-APB caused an increase in the percentage of cells in the S phase and a corresponding decrease in the G0/G1 phase in WT-231 but not in KO-231 (Figure 7C,D).

### 2.11. A Second Inhibitor, Gin Rd, Affected MDA-MB-231 Cells Similarly

**Figure 8:**
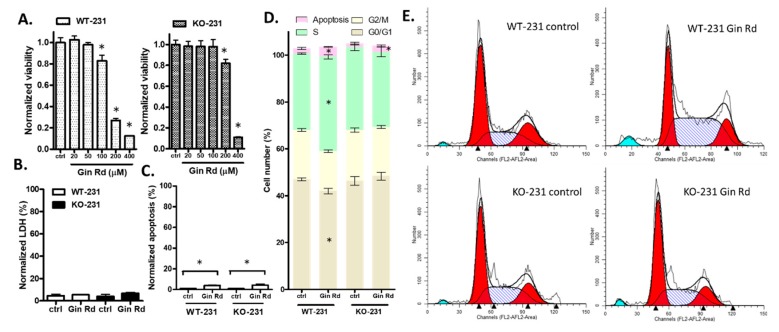
The effect of Gin Rd on TRPM7 knockout MDA-MB-231 cell biology. (**A**) Viability. Gin Rd (100 µM) suppressed viability in WT-231 only, while Gin Rd (200–400 µM) significantly suppressed viability in both WT-231 and KO-231, with a higher suppression rate in WT-231 than KO-231. Gin Rd (200 µM) was used in the subsequent experiments. (**B**–**E**) show data for cells exposed to 200 µM Gin Rd for 24 h. (**B**) LDH release. Gin Rd had no significant effect on LDH. (**C**) Apoptosis. Gin Rd slightly induced apoptosis in both cell lines. (**D**) Cell cycle analysis. The knock-out of TRPM7 did not affect the cell cycle. Gin Rd caused an increase in the percentage of cells in the S phase and a corresponding decrease in the G0/G1 phase in WT-231 but not in KO-231. (**E**) Representative images from the cell cycle assay of MDA-MB-231. Significant differences (*p* < 0.05) from control are indicated by “*”.

One of the concerns about using 2-APB is that it may have off-target effects. To help confirm the role of TRPM7 in cell viability, we also conducted viability, LDH, apoptosis, and cell cycle assays with a second inhibitor, Gin Rd. Although there is slight difference between controls in different batches of experiments, results showed that 400 µM Gin Rd inhibited the viability of both cell lines. However, at 200 µM, there was a distinct difference: the viability of WT-231 decreased by 73.3% and the viability of KO-231 decreased by only 18.5% (Figure 8A). Gin Rd (200 µM) was used in subsequent experiments. Gin Rd had no significant effect on LDH, but slightly induced apoptosis in both cell lines (Figure 8B,C). Cell cycle analysis showed that knock out of TRPM7 did not affect the cell cycle, but Gin Rd caused an increase in the percentage of cells in the S phase and a corresponding decrease in the G0/G1 phase in WT-231 but not in KO-231 (Figure 8D,E). To summarize, Gin Rd exerted a similar effect on the cell cycle as 2-APB did.

## 3. Discussion

Breast cancer is the most common type of cancer and the second most common cause of cancer mortality for females in the world [22]. The complexity of breast cancer development and the tolerance of breast cancer to chemotherapy spur the search for new targets for breast cancer treatment and the development of new treatment modalities. TRPM7 was recently considered as a potential target for breast cancer treatment. We utilized an online-based RNA expression analyzing web server, GEPIA [23], to analyze whether the level of expression of the TRPM7 gene in breast cancer affects the overall survival of patients. There is a tendency for worse outcomes when the TRPM7 gene is highly expressed (Figure S3). TRPM7 is found to be more highly expressed in some types of human cancer tissue compared to adjacent non-tumor tissue [24] and is thought to be associated with cancer development [8]. The effect of TRPM7 is complex because it affects cells both by regulating the flux of divalent cations into the cell and by regulating intracellular processes via the enzymatic activity of the kinase domain in its C-terminal region [25]. In this study, we explored how TRPM7 affects cell biology using two approaches: (1) overexpression of TRPM7 in a non-cancerous cell line and (2) knock-out of TRPM7 from a breast cancer cell line.

To determine the expression of the TRPM7 protein, we performed western blotting with seven TRPM7 antibodies from different sources. However, none of the antibodies proved to be reliable (data not shown). It is likely that other investigators have had the same problem because western blot evidence for TRPM7 protein is seldom reported. Instead, mRNA evidence for TRPM7 has been reported in many breast cancer cell lines, including T47D [26]. Using RT-QPCR, we confirmed that TRPM7 mRNA was expressed in MDA-MB-231, AU565, and T47D cells.

One concern for this study is that there is no ideal specific TRPM7 inhibitor. We screened several TRPM7 inhibitors, 2-APB, Gin Rd, and Waix A, but none of these inhibitors are specific to TRPM7: (a) 2-APB affects inositol 1,4,5-trisphosphate receptors, store-operated calcium channels and TRP channels, TRPV3 and TRPM7 [16,27]; (b) Gin Rd affects TRPM channels, acid-sensing ion channels (ASIC), and some cation channels [17,28]; (c) Waix A is a newly identified TRPM7 inhibitor [29,30]. Although Waix A does not inhibit TRPM2, TRPM4, TRPM6 or CRAC channels, its potency depends on the intracellular Mg^2+^ concentration and it is known to affect some cell lines that do not express TRPM7 [18,30]. We found that all three inhibitors preferentially suppressed the viability of HEK-M7 over WT-HEK, consistent with the fact that functional TRPM7 channels are critical for cell viability [19,20]. In our experiments, 2-APB inhibited the viability of HEK-M7 cells without affecting WT-HEK cells at concentrations of 100–200 µM (Figure 1B–D). We considered this as evidence that the effect of 2-APB, at this range of concentration, on HEK cells is primarily due to inhibition of TRPM7. A previous patch-clamp study showed that the IC_50_ of 2-APB suppressing TRPM7 is about 178 µM [31]. In our HEK cell system, we used two independent methods, in that acute exposure to 2-APB blocked the ion permeability of TRPM7 channels with an IC_50_ of about 120 µM (Figure 2B,D). WT-HEK cells showed very low TRPM7-like currents in patch-clamp recording (Figure S1), which indicated the relatively low functional TRPM7 in WT-HEK. These results suggest that 2-APB suppressed TRPM7 channels but had negligible effects on other divalent cation channels in HEK-M7 cells. Notably, 200 µM 2-APB also suppressed divalent cation influx through MDA-MB-231 cells to almost the same level as in KO-231 cells without 2-APB (Figure 6A). Therefore, 2-APB was selected as an inhibitor for studying TRPM7 channels in these cell lines, and the results were confirmed by a second inhibitor, Gin Rd.

Consistent with the results in HEK cells, 2-APB and Gin Rd preferentially inhibited the viability of WT-231 over KO-231. At high concentrations, both of the inhibitors suppressed the viability of KO-231 (Figure 6C), indicating off-target effects at high concentrations. Gin Rd, which is also not specific to TRPM7, was shown to induce apoptosis in TRPM7-expressing gastric and breast cancer cells [17]. Our results showed that Gin Rd affected the KO-231 with a similar pattern as 2-APB, except for a slight and nonselective induction of apoptosis (Figure 8). Although neither 2-APB nor Gin Rd are specific to TRPM7, their commonality is a similar effect on cell cycle, hence the inhibition of TRPM7 channels is likely to underlie this effect.

It has been reported that either the over-expression or deficiency of TRPM7 causes the death of HEK cells, but this effect is time- and Mg^2+^ concentration-dependent [13,19,20]. The HEK-M7 cell line in this study expresses two or three times lower levels of TRPM7 than the cells used in those studies and does not result in cell death [32]. In our experimental condition, the overexpression of TRPM7 increased the cell number in HEK cells (Figure 1A), and the knockout of TRPM7 in MDA-MB-231 decreased the cell number (Figure 6B), which supports the assertion that the TRPM7 function facilitates the growth of cells. Nevertheless, this effect seems to be very weak, as the significant difference in cell number occurred only after 2–3 days. Regarding the fact that the knockout of TRPM7 did not change the cell cycle (Figure 7C), it is possible that the cells developed a mechanism to compensate for the lack of TRPM7.

TRPM7 generally affects the growth of many types of cells, including bone cells [33], muscle cells [34], and many other cancer cell lines. In this study, we found that TRPM7 affected cells through the regulation of the cell cycle rather than cell death. In addition, the viability of cells with higher levels of TRPM7 was more susceptible to suppression by TRPM channel inhibitors. These results suggest that TRPM7 is conducive to cell proliferation. Interestingly, in HEK-M7, the 2-APB suppression rate decreased as cell density increased (Figure 3C), while in MDA-MB-231, this trend was not obvious (Figure 6E). HEK-M7 was subjected to cell–cell contact inhibition which suppresses cell proliferation. This suppression is mediated by the Hippo/YAP pathway and suppresses cyclin D/Cdk4–6 formation, which is a key component of the G1 phase [35]; thus, cells with high density are arrested in the G1 phase [36]. As shown by the cell cycle analysis, since TRPM7 inhibition also arrested cells in the G1 phase, the difference between cells with or without 2-APB was eliminated. However, as a cancer cell line, MDA-MB-231 was not subjected to cell–cell contact inhibition [37].

The permeability of TRPM7 for Mg^2+^ is higher than that for Ca^2+^, suggesting that Mg^2+^ might play a more important role in TRPM7 regulation. It is still controversial as to whether TRPM7 affects cells by changes in the ion permeability of the channel or changes in the enzymatic activity of the kinase domain [16]. Moreover, the TRPM7 channel’s kinase activity is essential for the TRPM7 channel function [16] and interacts with the store-operated calcium entry channel [38]. These all increase the difficulty to study the specific roles of TRPM7 in cancer cell proliferation. In the present study, the expression of TRPM7 in breast cancer cell lines was not affected by 2-APB (Figure 5B). In TRPM7 expressing cells (MDA-MB-231, AU565, T47D, and HEK-M7), the block of TRPM7 channels by 2-APB had little effect on cell death (LDH and apoptosis assay) but resulted in an increase in the percentage of cells in the S phase and a corresponding decrease in the G0/G1 phase. It is likely that the decrease in cell viability was caused by the inability to leave the S phase. A decreased expression of TRPM7 was reported to induce apoptosis in bladder cancer cells [39], but here we found that apoptosis was low in breast cancer cells and independent of the presence of TRPM7. In as much as the mechanism of TRPM7 regulation of the cell cycle is still unclear and may involve some cell cycle checkpoint signaling [40], we found that the channel function of TRPM7 promotes the activity of cells in the G1 and S phases. This is in agreement with a previous finding that the Ca^2+^ and Mg^2+^ influx mediated by TRPM7 channels improves the activities of cells in the S phase [8,41].

In summary, TRPM7 is expressed and plays a role in many breast cancers. The evidence provided in this study helps further define the role that TRPM7 plays in breast cancer cells. Our study contributes to the development of the TRPM7 study as a promising target against breast cancer. However, the direct link between the TRPM7 channel and the cell cycle is still missing and needs to be studied further.

## 4. Materials and Methods

### 4.1. Cell Lines and Cell Culture

Human kidney embryo cell line HEK293 (WT-HEK), breast cancer cell line MDA-MB-231 (WT-231), AU565, and T47D were obtained from ATCC^®^ (Manassas, VA, USA). The murine TRPM7 gene (GenBankTM accession number AF376052) overexpressing HEK293 clone (HEK-M7) was a gift from Dr. Loren Runnels, Robert Wood Johnson Medical School (Piscataway, NJ, USA) [32]. TRPM7 knock-out MDA-MB-231 cell line (KO-231) was obtained commercially from GenScript (Piscataway, NJ, USA) through GenCRISPRTM Technology and the knockout confirmed by genetic sequencing. The genetic sequencing shows a frameshift mutation at the TRPM7 gene (evidence can be provided upon reasonable request). HEK293 and MDA-MB-231 were cultured in Dulbecco’s Modified Eagle Media (DMEM) with 10% Fetal bovine serum (FBS), 10 mM piperazineethanesulfonic acid (HEPES), and 2% pen/strep. AU565 and T47D were cultured in Roswell Park Memorial Institute (RPMI)-1640 with 10% FBS, 10 mM, HEPES, and 2% pen/strep. All cell lines were cultured at 37 °C in a 5% CO_2_ humidified incubator and the medium was exchanged once per two days.

### 4.2. Drugs and Treatments

2-Aminoethyl diphenylborinate (2-APB) and tetracycline were purchased from Sigma-Aldrich (St. Louis, MO, USA). Ginsenoside Rd (Gin Rd) was purchased from Cayman Chemical Company (Ann Arbor, MI, USA). Waixenicin A was obtained from F. David Horgen’s lab at Hawaii Pacific University (Honolulu, HI, USA). The cell lines underwent serum starvation for 24 h before drug treatment. HEK-M7 was induced by 10 µg/mL tetracycline for 24 h before the starvation. All the assays were conducted after 24 h treatment, except for the patch-clamp and fluorescence quench assay.

### 4.3. MTT Assay

The cells were plated in 96-well plates (3–5 × 10^3^/well) for 12 h and were treated as described before [42]. Then, cells were incubated with 20 μL of 5 mg/mL MTT (Abcam, Cambridge, UK) for 2 h and the resulting formazan crystals were dissolved in dimethyl sulfoxide (200 μL). Absorbance was measured at 490 nm using the Thermo Scientific™ Multiskan™ (Waltham, MA, USA) FC Microplate Photometer. All the data were normalized with vehicle-treated control cells. The experiment was performed at least in triplicate and repeated three independent times.

### 4.4. Cell Counting

The cells were plated in 35 mm cell culture plates at different concentrations [43] 12 h prior to the experiment. When measuring the proliferation, the initial cell density was 0.5 × 10^5^ cells/mL. When measuring the effect of the drugs, the initial cell density was set at 0.5 × 10^5^, 1 × 10^5^, 2 × 10^5^ and 4 × 10^5^ cells/mL. Bright-field images were taken, and an aliquot of the cells was counted by CytoSMART Corning Cell Counter (Eindhoven, Netherlands).

### 4.5. Patch-Clamp Recording

Whole-cell currents were recorded with a patch-clamp amplifier (EPC 10 USB Single Patch Clamp Amplifier, HEKA Elektronik (Berlin, Germany) and PatchMaster software. The drugs were applied and removed using a multibarrel perfusion system (SF-77B Perfusion Fast-Step Translator, (Warner Instruments, Hamden, CT, USA)). The normal extracellular solution (ECS) contained (in mM) 140 NaCl, 25 HEPES, 1.3 CaCl_2_, 1.0 MgCl_2_, KOH (pH 7.3), 320–335 mOsm. The patch electrodes contained (in mM) 140 CsF, 7 NaCl, 10 HEPES and 11 EGTA, and the pH was adjusted to 7.3 using CsOH, 300 mOsm. The electrode was placed on the cell and gentle suction was applied to form a giga-ohm seal. More suction was applied to rupture the membrane patch, thus providing electrical access from the interior of the pipette to the intracellular space of the cell. Capacitance and series resistance compensation was performed to minimize the effects of series resistance on the applied voltage. The voltage was clamped at −80 mV, and a voltage ramp from −80 to +80 mV was applied over one second. Currents (normalized by whole-cell capacitance) were plotted as a function of voltage. At negative potentials, the current was linear, due to the seal resistance and/or voltage-independent channel activity. The data between −80 and 0 mV was fitted to a line, and this fit was used to subtract the linear component over the entire voltage range. This process revealed outwardly rectifying currents at positive potentials, as expected for TRPM7 channels. The rectifying currents at +80 were recorded and normalized by cell capacitance.

### 4.6. Fura-2AM-Based Quench Assay

The cells (50,000–60,000 cells/well) were plated in 96-well plates and TRPM7 expression was induced 3 h post-plating by tetracycline in HEK-M7. The culture medium was completely removed after 18 h post-induction and replaced with fura-2 loading-buffer: 2 mM fura-2-acetoxymethyl ester (Abcam, Cambridge, UK) in ECS. Following incubation (60 min at 37 °C), the loading buffer was removed, and the cells were washed once with ECS before the addition of fresh ECS as the assay buffer. The plates were then transferred to a pre-warmed (37 °C) fluorescence plate reader ((PerkinElmer, Waltham, MA, USA) Victor X3). In the quench assay, cells were incubated with test substances or vehicles for 5 min after loading. Vehicle-receiving induced HEK-M7 cells served as a positive control for the activation of a TRPM7-mediated Mn^2+^-influx, while wells containing vehicle-receiving WT-HEK served as negative controls to define the nonspecific flux of Mn^2+^. The Ca^2+^-independent fluorescence (excitation 360 nm; emission 510 nm) of fura-2 was monitored following the addition of 10 mM MnCl_2_. The method was tested by Waix A on HEK-M7 (Figure S4) and the dose–response curve was similar to the result reported in [30].

### 4.7. Quantitative Real Time-PCR

The isolation of the total RNA was done with the RNeasy Mini kit (Qiagen, Germantown, MD, USA). First-strand DNA was generated from 0.5 μg of the total RNA using oligo (dT) 15 and reverse transcriptase SuperScript IV (Invitrogen, Carlsbad, CA, USA) at a reaction volume of 20 μL. Human TRPM7 and human glyceraldehyde-3-phosphate dehydrogenase (GAPDH) primer pairs were synthesized by the DNA Sequencing Facility at Stony Brook University. The primers used for RT-PCR [9] were TRPM7 forward (5’-TAGCCTTTAGCCACTGGAC-3’), TRPM7 reverse (5’-GCATCTTCTCCTAGATTTGC-3’), GAPDH forward (5’-GAAGGTGAAGGTCGGAGTC-3’), and GAPDH reverse (5’-GAAGATGGTGATGGGATTTC-3’). Quantitative real time-PCR was done with PowerUp™ SYBR™ Green Master Mix (Thermo, Beverly, MA, USA) using ViiA 7 Real-Time PCR System (Applied Biosystems, Foster City, CA, USA). Negative control in the PCR reaction was done by replacing cDNA with ultrapure water. The protocol for PCR amplification consisted of denaturation at 94 °C for 3 min, 35 cycles of denaturation at 94 °C for 30 s, annealing at 57 °C for 15 s, and extension at 72 °C for 30 s. The data were normalized using the 2^△△CT^ method.

### 4.8. LDH Assay

The cells were plated in 96-well plates (3–5 × 10^3^/well) for 12 h and were treated as described before. LDH in the medium was then detected using the Cytotoxicity Detection Kit (Sigma-Aldrich, St. Louis, MO, USA). LDH assays were carried out according to the manufacturer’s instructions. The LDH level in cells exposed to Triton X-100 was considered as 100% to normalize the results. The absorbance was measured at 490 nm using the Multiskan™ FC Microplate Photometer.

### 4.9. Apoptosis Assay

The cells were plated in 96-well plates (3–5 × 10^3^/well) for 12 h and were treated as described before. The apoptosis assays were then performed using Cell Death Detection ELISA plus (Roche, Indianapolis, IN, USA), which monitors DNA fragmentation. The absorbance was measured at 490 nm using the Multiskan™ FC Microplate Photometer. We prepared a positive control for each cell line by inducing cell death through incubating cells at 55 °C for 20 min.

### 4.10. Cell Cycle Flow Cytometry

The cell cycle was analyzed with flow cytometry and propidium iodide. After treatment, the cells were washed with cold Phosphate-Buffered Saline (PBS) and resuspended at 1 × 10^6^/mL. The cells were fixed by adding an equal volume of cold absolute ethanol and were incubated for at least two hours at 4 °C. The cells were washed with cold PBS, and stained with propidium iodide (0.1% Triton X-100, 0.2 mg/mL DNAse-free RNAse A, 0.02 mg/mL in cold PBS) at 37 °C for 15 min. BD FACSCalibur was used to acquire data, which was then analyzed by FlowJo (Version 9.3.2, Ashland, OR, USA) using the Dean–Jett–Fox fit.

### 4.11. Statistics

Experiments were repeated at least three times. Means and standard deviations are shown in the figures. A *T*-test or ANOVA was used to assess the significance (*p* < 0.05). Dunnett’s post hoc tests were used to test the difference between groups. GraphPad Prism (version 6) was used to calculate statistics.

## 5. Conclusions

Our results suggest that TRPM7 regulates the viability of breast cancers and is a potential therapeutic target against breast cancer. The direct link between the TRPM7 channel and the cell cycle is still missing and needs to be studied further.

## Supplementary Materials

The following are available online at https://www.mdpi.com/2072-6694/12/1/131/s1, Figure S1: Representative TRPM7-like current from a whole-cell patch-clamp experiment with a WT-HEK cell, Figure S2: Normalized quench levels averaged over 320–350 s in MDA-MB-231 (*n* = 3), Figure S3: Effect of high expression of TRPM7 gene in breast cancer on Overall Survival of clinical patients, Figure S4: The suppression of Waxi A on TRPM7 channels.
