# Supplementary material for: The Role of Transient Receptor Potential Melastatin 7 (TRPM7) in Cell Viability: A Potential Target to Suppress Breast Cancer Cell Cycle
Source: Cancers (Basel). 2020 Jan 4;12(1):131. doi: 10.3390/cancers12010131 (PMC7016641; doi:10.3390/cancers12010131)
Supplement: Supplementary file 1 [file cancers-12-00131-s001.pdf]

Supplementary Materials:

The Role of Transient Receptor Potential Melastatin 7 (TRPM7) in Cell Viability: A Potential Target to Suppress Breast Cancer Cell Cycle

Hengrui Liu †, James P. Dilger and Jun Lin *

1. Patch-clamp recording

The method of patch-clamp recording was described previously in the Materials and Methods of the main part of the paper.

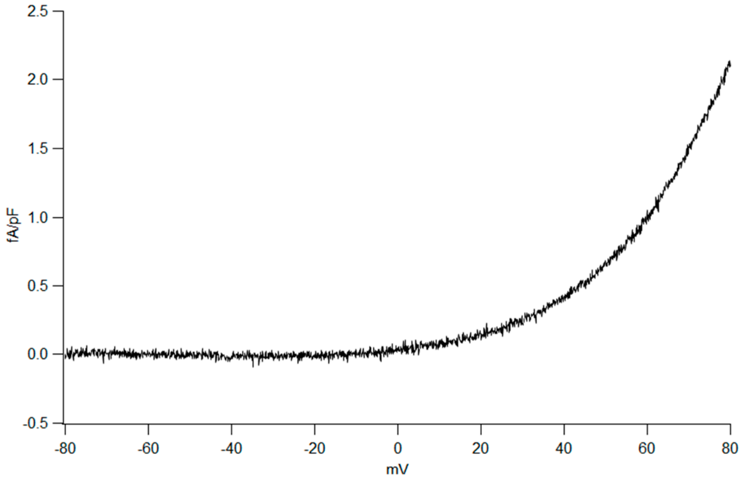

Figure S1. Representative TRPM7-like current from a whole-cell patch-clamp experiment with a WT-HEK cell. The voltage was clamped at -80 mV, then ramped from -80 to +80 mV. The outwardly rectifying current at positive potentials was suppressed by a perfusing solution containing 2-APB and completely recovered by perfusing 2-APB-free solution. There was a very low detectable rectifying current in WT-HEK cells.

2. Normalized quench levels averaged over 320-350 s in MDA-MB-231.

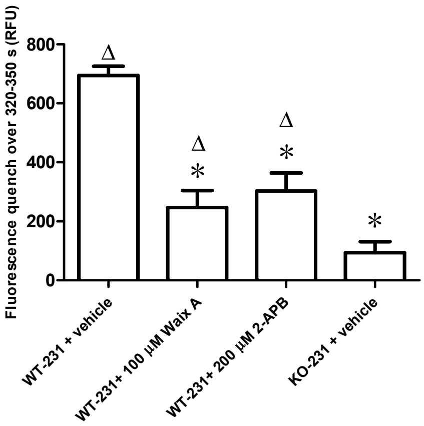

Figure S2. Normalized quench levels averaged over 320–350 s in MDA-MB-231 (n = 3). Significant differences from "WT-231 + vehicle" and "KO-231 + vehicle" are indicated by * $p < 0.05$ and $^{\Delta}$ $p < 0.05$ respectively.

3. Genetic Data Analysis

To associate our study with clinical breast cancer treatment, an online-based RNA expression analyzing web server, GEPIA, was used to analyze the RNA sequencing expression data of colon tumors and normal colon samples from the TCGA and the GTEx projects. The effect of high expression of the TRPM7 gene in breast cancer on Overall Survival of clinical patients was analyzed. Results showed that there is no statistical significance, but there is a tendency to have a worse outcome with high expression of TRPM7, which is in agreement with our conclusion.

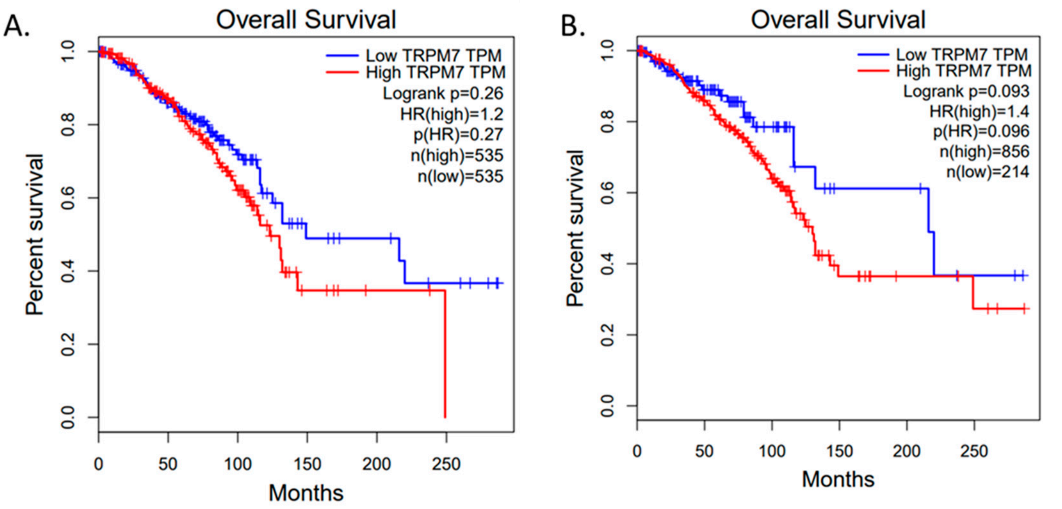

Figure S3. Effect of high expression of TRPM7 gene in breast cancer on Overall Survival of clinical patients. (**A**) Samples with expression level higher and lower than 50% threshold are considered as the high- and low- expression cohort respectively. (**B**) Samples with expression level higher and lower than 20% threshold are considered as the high- and low- expression cohort respectively.

4. Waxi A Fura-2AM-Based Quench Assay

The method of fluorescence quench assay is described in the Materials and Methods of the main part of the paper.

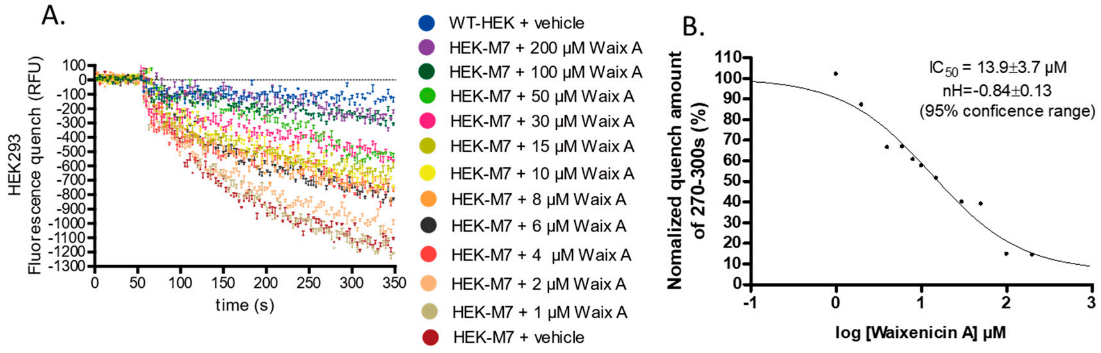

Figure S4. The suppression of Waxi A on TRPM7 channels. (**A**) Results from experiments using Mn^{2+} quenching of Fura-2AM fluorescence. Waxi A suppressed fluorescence quenching by blocking entry of Mn^{2+} via TRPM7 channels. Fluorescence quenching in WT-HEK cells indicates a flux of Mn^{2+} through pathways other than TRPM7 channels. (**B**) Normalized quench levels averaged over 320–350 s for three experiments. The IC_{50} and Hill slope of quench blockade are 13.9 ± 3.7 μM and −0.84 ± 0.13 respectively (95% confidence range).
